# Methylergometrine-Induced Myocardial Infarction in the Setting of a Cesarean Delivery

**DOI:** 10.7759/cureus.20068

**Published:** 2021-12-01

**Authors:** Mohamed Fayed, Branden Buffington, Rowaa Ibrahim, Ami Y Attali, Joshua Younger

**Affiliations:** 1 Anesthesiology, Perioperative Medicine and Pain Management, Henry Ford Health System, Detroit, USA; 2 Obstetrical Anesthesiology, Henry Ford Health System, Detroit, USA

**Keywords:** non-stemi, non-st-elevation myocardial infarction, methylergonovine, uterine atony, obstetric anesthesia, obstetric hemorrhage, methylergometrine, postpartum pulmonary edema, cardiogenic pulmonary edema, c-section

## Abstract

A 30-year-old female with no significant past medical history presented to our labor and delivery ward for induction of labor. Due to failure to progress, she was proceeded to cesarean delivery. Intraoperatively, it was noted that her uterus was hypotonic; she required supplemental methylergometrine to control the bleeding from the uterine atony. However, within three minutes of intramuscular (IM) administration, she complained of chest pain. She then subsequently developed pulmonary edema in the postoperative care unit, which required supplemental oxygen. She was found to have elevated troponin and brain natriuretic peptide (BNP), along with radiologic features of fluid overload suggestive of congestive cardiac failure, which all lead to the diagnosis of non-ST myocardial infarction. The patient had a normal computed tomography (CT) pulmonary angiogram, echocardiogram, and serial electrocardiograms (ECGs). She was successfully discharged from the hospital on postoperative day 4 with resolution of her symptoms and improving cardiac enzymes. Cardiology outpatient follow-up was arranged.

## Introduction

Methylergometrine is a semisynthetic analog of ergometrine that has a long history of being used to prevent or control postpartum hemorrhage [1,2]. Pharmacokinetic studies following intravenous injection have shown that methylergometrine is rapidly distributed from plasma to peripheral tissues within two to three minutes [3]. Methylergometrine prevents or controls postpartum hemorrhage through uterotonic effects at the inner layer (archemyometrium) of the uterus, causing muscle contraction and direct vasoconstriction of the uterine blood vessels [1]. It achieves its vasoconstrictive effects through partial antagonism of the alpha-adrenergic receptors in the uterine blood vessels [1]. It is not well understood how it achieves its uterotonic effects, but it is believed to be through its interaction with the Ca2+ receptors of the smooth muscle in the archemyometrium [1]. Methylergometrine has multiple side effects, including asthma exacerbation, nausea, vomiting, arrhythmias, hypertension, and coronary artery spasm [2].

There are six cases documenting methylergometrine-induced myocardial infarction in the setting of obstetric procedures [4-9]. The patients range in age from 22 to 38 years old. In a majority of the cases, there were no relevant cardiac risk factors or significant past medical history. The onset of symptoms ranged from 15 minutes after administration of methylergometrine to five days later [4-7,9]. The most common presenting symptom was chest pain [5,7].

We report the novel case of a patient who developed chest pain within three minutes of intramuscular (IM) injection of methylergometrine while under subarachnoid block for a caesarian section with subsequent development of pulmonary edema in the postoperative care unit. Although coronary artery vasospasm is a cited side effect of methylergometrine [10,11], there is limited literature in the clinical setting reporting immediate chest pain within three minutes of administration of methylergometrine.

## Case presentation

A 30-year-old female with no significant past medical history presented for induction of labor for her first pregnancy at 40 weeks of gestation with an uneventful pregnancy. An epidural catheter was inserted at the L4-L5 level, and intermittent boluses of bupivacaine 0.0625% with fentanyl 2 mcg/mL were used to achieve labor analgesia. To augment labor, oxytocin infusion was started at 2 milliunits/minute and gradually up-titrated to 30 milliunits/minute. After 12 hours of attempting to induce labor, a decision was made to proceed to cesarean delivery due to inadequate cervical dilatation. Epidural anesthesia was achieved using 17 mL of lidocaine 1.5% with epinephrine 1:200,000. Sensory block was at the level of T4 dermatome. The patient then required intermittent boluses of 5 mL of lidocaine 1.5% with epinephrine 1:200,000 every 15 minutes to maintain T4 level. The patient was hemodynamically stable with a heart rate ranging from 80 to 90 beats per minute and blood pressure ranging from 120/50 to 130/70 mmHg. The patient did not require medications to support her blood pressure.

The cesarean section proceeded without incident, and the newborn was successfully delivered 30 minutes after starting epidural anesthesia. The placenta was delivered spontaneously with standard 20 units of oxytocin infusion. During the closure of the hysterotomy, uterine atony was noted despite an additional infusion of 20 units of oxytocin. Five and seven minutes later, additional doses of IM injection of methylergometrine 0.2 mg followed by IM injection of carboprost 250 mcg were given, respectively. The uterotonic choice was recommended by the obstetrician. The patient then reported to her husband and the anesthesia provider that she felt chest pain three minutes after methylergometrine injection and peaked after 20 minutes. A five-lead continuous electrocardiogram (ECG) monitor was negative for ST changes during this episode, and the chest pain peaked at 20 minutes. Her vital signs were stable with a heart rate ranging from 80 to 100 beats per minute, blood pressure ranging from 120/60 to 135/70 mmHg, and oxygen saturation above 96% on 2 L nasal cannula. Chest auscultation did not show evidence of wheezes. She additionally experiences an intraoperative episode of emesis and complained of shortness of breath after the chest pain. The estimated blood loss at the end of the surgery was 790 mL.

**Figure 1:**
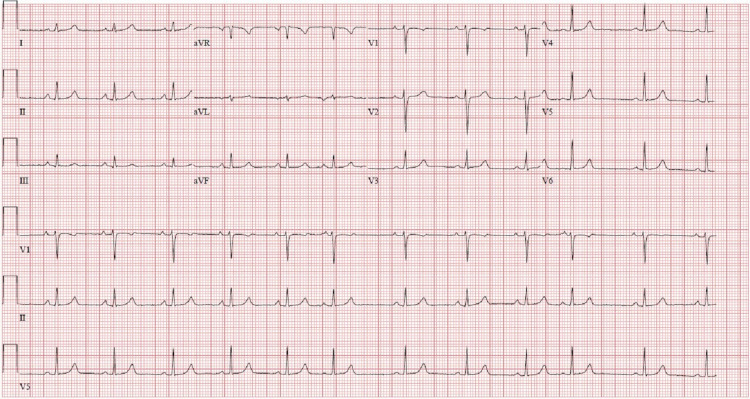
ECG tracing of our patient. ECG: electrocardiogram

**Figure 2:**
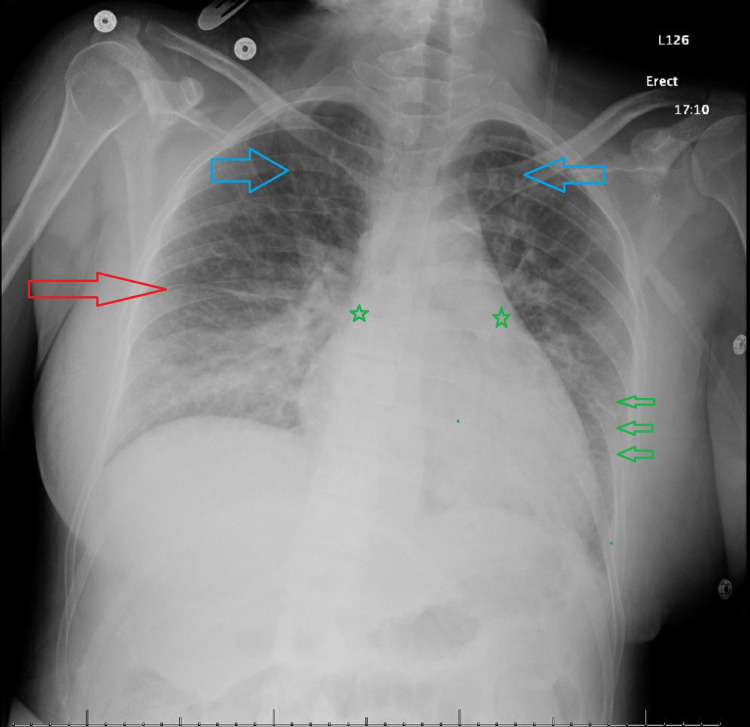
Chest X-ray of our patient showing the picture of the pulmonary edema. Red arrow: fluid in the transverse fissure; green arrows: Kerley B lines; blue arrows: cephalization of blood vessels; green stars: fullness in the hilum

In the postanesthesia care unit, the patient complained of worsening shortness of breath with an associated drop in her oxygen saturation to a nadir of 90% on 2 L oxygen via nasal cannula. The patient then required escalation of oxygen therapy to 8 L via a face mask to maintain oxygen saturation of more than 93%. Chest auscultation showed decreased air entry over lung bases with fine basal crepitations. An ECG was performed, which showed normal sinus rhythm without evidence of ischemic changes (Figure 1). A chest X-ray was ordered, which showed a picture of fluid overload suggestive of heart failure (Figure 2). Laboratory workup showed normal liver function tests, complete blood count including white cell and platelet counts, electrolyte tests, and renal function tests. The initial troponin level was elevated at 47 ng/L and then peaked to 60 ng/L (normal range: <19 ng/L) with an initial brain natriuretic peptide (BNP) of 1050 pg/mL (normal range: <50 pg/mL).

**Figure 3:**
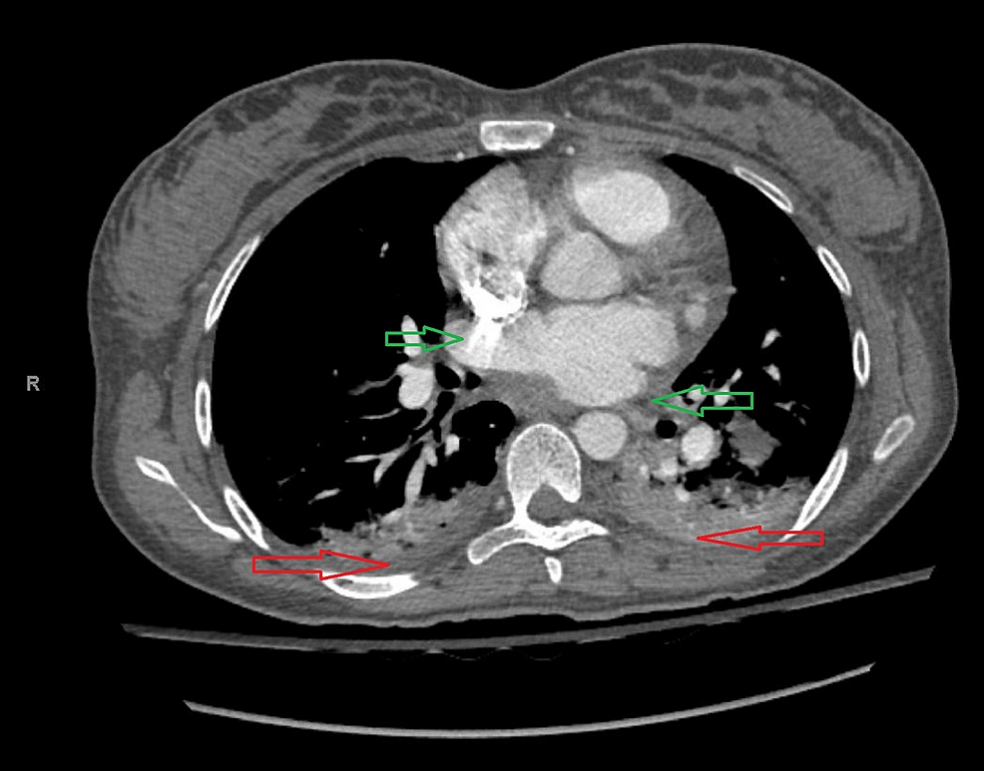
CT pulmonary angiogram showing bilateral pleural effusions with no evidence of pulmonary embolism. CT: computerized tomography; red arrows: pleural effusions; green arrows: pulmonary arteries

The patient was started on diuretics using furosemide 80 mg intravenous as the initial dose and then intermittent doses of 20 mg intravenously, and pain control using multimodal pain control regimen. The cardiology team was consulted, and the impression was either NSTEMI secondary to methylergometrine or peripartum cardiomyopathy. Additional investigations were organized, including CT pulmonary angiogram and transthoracic echocardiogram. CT pulmonary angiogram did not show evidence of clots in pulmonary arteries; however, it showed bilateral pleural effusion and pulmonary edema (Figure 3).

Transthoracic echocardiogram on the next day showed normal left and right ventricular systolic and diastolic functions, without evidence of valve abnormalities or regional wall motion abnormalities. Patient symptoms gradually improved in the next few days. Repeated ECGs were normal. Given her symptom improvement and that the patient was able to ambulate without chest pain, the cardiology team decided to follow her up in the outpatient clinic with a possible exercise stress test. The patient was discharged home, and she is following up with the outpatient clinic at regular intervals.

## Discussion

Our patient started having chest pain and shortness of breath within a few minutes after methylergometrine injection while she was under epidural anesthesia, which got worse in the postanesthesia care unit. Another differential diagnosis of this presentation includes peripartum cardiomyopathy, pulmonary embolism, severe preeclampsia, postpartum fluid overload, pneumonia, bronchospasm, high level of epidural anesthesia, and myocardial ischemia.

Our patient did not have a history of infective symptoms. Her laboratory workup, including white cell count, chest X-ray, and CT scan, were not suggestive of any infective etiology. Pulmonary embolism was ruled out given the CT pulmonary angiogram findings and transthoracic echocardiogram features. Severe preeclampsia and hemolysis, elevated liver enzymes, and low platelet count (HELLP) syndrome were also ruled out given the laboratory workup findings with lack of proteinuria, normal platelet counts, and liver function tests. Bronchial asthma exacerbation was also ruled out given her auscultatory findings. Peripartum cardiomyopathy was included in the differential diagnosis; however, prior to delivery, the patient did not have symptoms suggestive of heart failure, her transthoracic echocardiogram features did not show evidence of heart failure, and her symptoms improved after medical therapy. The presence of chest pain with elevated troponin levels in the context of methylergometrine injection supported the diagnosis of myocardial infarction.

Myocardial infarctions related to pregnancy are rare events with 3-10 cases/100,000 deliveries being reported in the literature [12]. Myocardial infarctions in the setting of methylergometrine use are even rarer findings. To our knowledge, there are only six cases documenting such events [4-9]. The patients ranged in age from 22 to 38 [1-6]. In a majority of the cases, there were no relevant cardiac risk factors or significant past medical history. The onset of symptoms ranged from 15 minutes after administration of methylergometrine to five days later [4-8]. Two cases reported onset of symptoms in 15 minutes and 3 hours; both were post-IM injections [6,13]. This was compared with oral methergine, in which chest pain started at the third to fifth day [4-5,9]. Our patient had onset in three minutes and peaked at 20 minutes, which coincide with the available pharmacokinetics of methylergonovine [3]. Based on the manufacturer data, the onset of action after IV administration is immediate compared with 2-5 minutes after IM administration and 5-10 minutes after oral administration [3]. In pharmacokinetic studies following 0.2 mg IM injection, a mean peak plasma concentration of 5918 ± 1952 pg/mL was observed at 24 ± 12 minutes [3]. The most common presenting symptom was chest pain [5-9]. Additionally, a majority of the cases were accompanied by ECG changes that showed ST-segment elevation [4-6]. Coronary angiography was also performed in two of the cases showing significant stenosis of coronary arteries [4,5]. Echocardiography was performed in two of the cases showing left ventricle dyskinesia [5,7]. There was also one case that reported subsequent mortality following methylergometrine use [8]. We were also able to find two cases that mentioned pulmonary edema and dyspnea following administration of methylergometrine, but with the lack of chest pain or ECG changes [9,10].

This case report highlights the importance of considering the benefits and risks of methylergometrine in managing every case. There is a very small risk of myocardial injury and infarction; however, clinicians should not delay administering methylergometrine during life-threatening bleeding secondary to uterine atony. As a matter of fact, delaying in the administration of uterotonic medications was implicated as an important risk factor in the development of severe postpartum hemorrhage with a resultant increase in maternal morbidity and mortality [14]. Other pharmacological alternatives to methylergometrine have their own side effect profile, e.g., carboprost and misoprostol [15]. Further, it is not uncommon for patients to require treatment with more than one uterotonic drug to achieve adequate uterine tone.

## Conclusions

Myocardial infarction can happen in the setting of methylergometrine use. Chest pain is a common presenting symptom especially if the patient was under regional anesthesia. This pain can happen within a few minutes of administration. Early recognition and prompt treatment is the key to management. It is worth mentioning that the risk of myocardial infarction is extremely low, and this should not hinder clinicians from administering methylergometrine during left-threatening bleeding secondary to uterine atony.
